# Classification of barley U-box E3 ligases and their expression patterns in response to drought and pathogen stresses

**DOI:** 10.1186/s12864-019-5696-z

**Published:** 2019-04-29

**Authors:** Moon Young Ryu, Seok Keun Cho, Yourae Hong, Jinho Kim, Jong Hum Kim, Gu Min Kim, Yan-Jun Chen, Eva Knoch, Birger Lindberg Møller, Woo Taek Kim, Michael Foged Lyngkjær, Seong Wook Yang

**Affiliations:** 1 Plant Biochemistry Laboratory, Department of Plant and Environmental Sciences, University of Copenhagen, Thorvaldsensvej 40, DK-1871 Frederiksberg C, Copenhagen, Denmark; 2 Department of Systems Biology, College of Life Science and Biotechnology, Yonsei University, Seoul, 120-749 Korea; 3 Institute of Life Science and Biotechnology, Yonsei University, Seoul, South Korea; 4 Samsung Medical Center, 81 Irwon-Ro Gangnam-gu, Seoul, Korea

**Keywords:** Barley, *Hordeum vulgare*, Ubiquitin proteasome system (UPS), Biotic stress, Abiotic stress

## Abstract

**Background:**

Controlled turnover of proteins as mediated by the ubiquitin proteasome system (UPS) is an important element in plant defense against environmental and pathogen stresses. E3 ligases play a central role in subjecting proteins to hydrolysis by the UPS. Recently, it has been demonstrated that a specific class of E3 ligases termed the U-box ligases are directly associated with the defense mechanisms against abiotic and biotic stresses in several plants. However, no studies on U-box E3 ligases have been performed in one of the important staple crops, barley.

**Results:**

In this study, we identified 67 putative U-box E3 ligases from the barley genome and expressed sequence tags (ESTs). Similar to Arabidopsis and rice U-box E3 ligases, most of barley U-box E3 ligases possess evolutionary well-conserved domain organizations. Based on the domain compositions and arrangements, the barley U-box proteins were classified into eight different classes. Along with this new classification, we refined the previously reported classifications of U-box E3 ligase genes in Arabidopsis and rice. Furthermore, we investigated the expression profile of 67 U-box E3 ligase genes in response to drought stress and pathogen infection. We observed that many U-box E3 ligase genes were specifically up-and-down regulated by drought stress or by fungal infection, implying their possible roles of some U-box E3 ligase genes in the stress responses.

**Conclusion:**

This study reports the classification of U-box E3 ligases in barley and their expression profiles against drought stress and pathogen infection. Therefore, the classification and expression profiling of barley U-box genes can be used as a platform to functionally define the stress-related E3 ligases in barley.

**Electronic supplementary material:**

The online version of this article (10.1186/s12864-019-5696-z) contains supplementary material, which is available to authorized users.

## Background

**Fig. 1:**
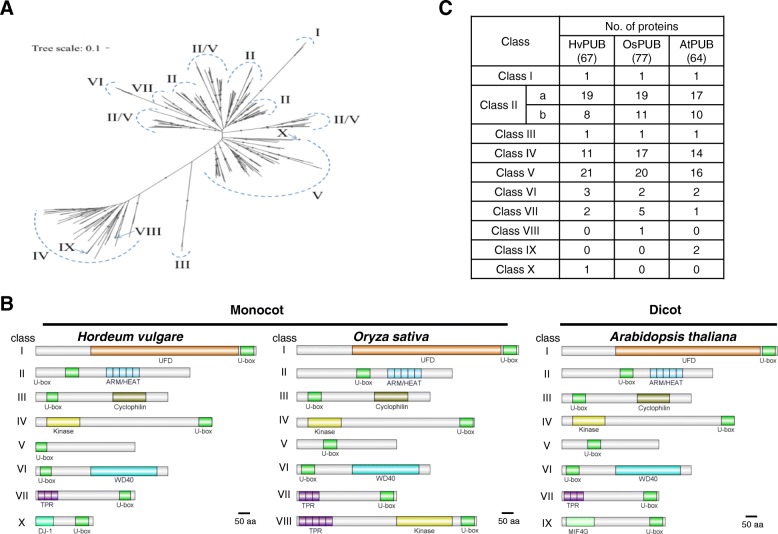
Phylogenetic identification and domain structures of the 67 *PUB* genes in barley. **a**. Full-length amino-acid sequences of *PUB* genes in Arabidopsis, rice and barely were analyzed using the Clustal X2 software. The tree was constructed by neighbor-joining method after bootstrap analysis for 1000 replicates [11]. **b**. Domain structures of the 67 *PUB* genes into 8 different classes. Green box, U-box domain; brown box, UFD2 UB chain assembly domain; sky blue box, ARM repeat domain; yellow box, kinase domain; cyan box, WD40 protein interaction domain; violet box, TPR; light green box, DJ-1 domain. C. Domain organization of *PUB* genes in Arabidopsis, rice and barley

The ubiquitin proteasome system (UPS) orchestrates turnover of a large number of proteins in eukaryotic cells and thereby regulates cellular responses to external and internal stimuli while maintaining house-keeping functions [1, 2]. The UPS is composed of three specific enzyme-types 1) ubiquitin-activating E1 enzymes, 2) ubiquitin-conjugating E2 enzymes and 3) ubiquitin E3 ligase enzymes. Through multiple ubiquitination cycles, specific proteins are targeted to the proteasome for degradation [3–5]. In plants, the importance of the UPS system is exemplified in Arabidopsis, where about 6% of the Arabidopsis genome or about 1600 genes encode core components of the UPS, including two E1 enzymes, at least 37 E2 enzymes and approximately 1.400 E3 ligases [1]. In general, the conjugation of ubiquitin(s) to a specific target protein is determined by the type of E3 ligase. The E3 ligases are classified into three families, the HECT-type, RING-type, and U-box-type E3 ligases, according to their functional domains. Among those, U-box-type E3 ligase is the smallest family with approximately 60 members in Arabidopsis [6–8]. The U-box containing proteins have been assigned as PLANT U-box (PUB) enzymes. All the defined PUB proteins in Arabidopsis and rice were named by consecutively numbering after the term PUB, except for the Arabidopsis U-box protein CHIP (Carboxyl terminus of HSC70-interacting protein) [9]. Based on the sequence of 63 identified Arabidopsis *At*PUB proteins and 77 rice *Os*PUB proteins, the proteins were assigned into nine different PUB classes according to their domain characteristics [8, 10] (Fig. 1c). Class I members (1 Arabidopsis; 1 rice) are homologues of the yeast *UFD2* (ubiquitin fusion degradation protein 2) which contains a UFD2 domain, known to interacts with the AAA family ATPase CDC48 protein [12]. Class II members (29 Arabidopsis; 28 rice) possess a variable number of Armadillo repeats (ARM) in their C-termini. These are thought to form an α-solenoid structure that might constitute a protein interaction domain [12–14]. Class III members (12 Arabidopsis; 16 rice) are suggested to possess a GKL motif (a conserved Glycine (G), Lysine (K)/Arginine (R) residues and its leucine rich residues) located close to the C-terminus [10, 12]. Class IV members (16 Arabidopsis; 9 rice) possess a serine/threonine kinase domain at the C-termini. Class V members (7 Arabidopsis; 8 rice) are characterized as PUB proteins without any additional recognizable domains [12]. Class VI members (2 Arabidopsis; 2 rice) possess a WD40 domain, which constitutes a well-known protein-to-protein interaction motif. Class VII members (1 Arabidopsis; 6 rice) contain a tetratrico-peptide repeat (TRP) domain, which has been shown to mediate protein-protein interactions [10]. Class VIII is rice specific and contains only one member, which in addition to the U-box domain possesses a TRP domain and a kinase domain. Class IX is Arabidopsis specific and contains two members with a MIF4G-type domain (Fig. 1b). The U-box E3 ligase family in grapevine [15] and Medicago [16] has been separated into classes similar to those in Arabidopsis and rice based on the domain structures present.

Functional analyses of PUB genes have mainly been conducted in Arabidopsis and rice, and document that the encoded PUB proteins play important roles in plant adaptation and response to many environmental stresses, including drought and microbial attack. For instance, the ubiquitination pathway has been implicated in both ABA-dependent and ABA-independent drought responses. Arabidopsis *AtPUB18* and *AtPUB19* are strongly up-regulated in response to abscisic acid (ABA) and mutation studies showed that the encoded proteins act as negative regulators of ABA-mediated drought responses [17] whereas the proteins *At*PUB22 and *At*PUB23 were shown to act as negative regulators of ABA-independent drought responses [18]. Arabidopsis *At*PUB18 and *At*PUB19 were also suggested to function as regulatory components in salt inhibited germination [19] and *At*PUB30 has been suggested to function as a negative regulator of salinity tolerance because loss of function mutants exhibited increased salt stress tolerance in the germination stage [20]. Rice, *Os*PUB2 and *Os*PUB3 apparently interact to form heterodimeric complexes and are involved in positive regulation of low temperature stress [21]. Phosphate starvation leads to strong up-regulation of rice *OsUPS* (*OsPUB41*), suggesting an important role of *Os*PUB41 in the Pi signaling pathway [22]. Several PUBs also play distinctive roles affecting plant growth and development. Arabidopsis SAUL1 (*At*PUB44) controls leaf senescence and enhances cell death in different tissues [23], whereas *At*PUB4 functions as a global regulator of asymmetric cell divisions and cell proliferation during root development [24] and rice TUD1 (*Os*PUB75) regulates brassinosteroid-mediated growth [25].

Some PUBs have been implicated in both abiotic and biotic stress responses. Together *At*PUB24, *At*PUB22 and *At*PUB23 are involved in PAMP-triggered immunity (PTI) towards microbials [26]. Likewise, rice SPL11 (*Os*PUB11) and its Arabidopsis orthologs *At*PUB12 and *At*PUB13 were shown to negatively regulate innate immunity and defense responses [27–29] by their ability to ubiquitinate the receptor-like kinase FLS2 (Flagellin Sensing 2) protein after bacterial infection. *Os*PUB11 ubiquitinates SPIN6 (a Rho GTPase-activating protein) controlling disease resistance signaling during both fungal and bacterial infection [27–29]. *Os*PUB44 positively regulates peptidoglycan- and chitin-triggered immunity and resistance to the bacterium *Xanthomonas oryzae* [30] and *Os*PUB15 interacts with the receptor-like kinase PID2 to regulate cell death and immunity against rice blast [31]. *At*PUB17 is a functional homolog of tobacco ACRE276, improving race-specific resistance against Avr9 from the pathogenic fungus *Cladosporium fulvum* and against the Gram-negative bacterium *Pseudomonas syringae* [32]. The potato homolog *St*PUB17 was shown to promote specific immune pathways triggered by *Phytophthora infestans* [33] suggesting a conserved function as positive regulators of cell death and defense for these Class II ARM repeat E3 ligases. Beside the rice PUB genes, only two other cereal PUB genes have been functionally characterized. Wheat *Ta*PUB1 was shown to modulate drought stress responses by modulating the antioxidant capability [34] and CMPG1-V from the diploid wheat relative *Haynaldia villosa* L. was shown to increase resistance against the powdery mildew fungus [35].

In this study, we have identified the members of the PUB protein family in barley based on the published high-quality reference genome sequence of barley (*Hordeum vulgare*) [36]. Using the available annotation, Hidden Markov Model genomic analysis and blast searches with Arabidopsis and rice PUB protein sequences we identified 67 *HvPUB* genes. Sequence alignments, domain patterns and phylogenetic analyses of the barley, Arabidopsis and rice PUB proteins revealed that the previous classification of PUB genes in Arabidopsis and rice has an ambiguity in the grouping of the Class III ligases [8]. We propose a re-classification of the Arabidopsis, rice and barley PUB proteins into 10 Classes according to their functional domains using NCBI CDD (conserved Domain Database) and InterPro protein domain predictions. The potential involvement of the predicted *HvPUB* genes in abiotic and biotic stress responses was investigated by analyzing public available full length cDNA and EST libraries and by expression profiling of the *HvPUB* genes under drought stress or during attempted infection by the powdery mildew fungus.

## Results

### Identification of U-box E3 ligase encoding genes in the barley genome

Based on the published high-quality reference genome sequence of barley (*Hordeum vulgare*) [36], BLAST searches using full length cDNA sequences and ESTs encoding U-box-type E3 ligases in Arabidopsis and rice as query sequences, 67 U-box E3 ligase encoding genes were predicted in barley (Fig. 1a, c). In agreement with current terminology the genes were termed *HvPUB* genes (Table 1). Mapping the genome loci onto the chromosomes shows that the *HvPUB* genes are distributed between all the chromosomes (Table 1). In a few places, two or more *HvPUB* genes are arranged tandemly or closely clustered together. *HvPUB11/12* locate in tandem and have 100% identical sequences, and this is also true for *HvPUB58/59*, indicating recent gene-duplications. However, the gene pairs such as *HvPUB6/43/52*, *HvPUB13/25*, *HvPUB15/16* and *HvPUB28/29* are different. Even though the genes map cluster together, they are not directly related, indicating that this clustering could be the result of evolutionary selective forces.

**Table 1:** List of 67 named barley *PUB* genes with their classification, genome locus, and the number of matching ESTs from libraries categorized as originated from either abiotic- or biotic stress conditions or from vegetative or generative tissue

Class	Name	Gene ID	Chromosome position	No. EST from library conditions			
				Abiotic	Biotic	Generative	Vegetative
				stresses			
I	HvPUB1	HORVU7Hr1G108540	chr7H:625630612–625,636,687	3		18	14
IIa	HvPUB2	HORVU0Hr1G020210	chrUn:105982242–105,985,875	14		4	3
IIa	HvPUB3	HORVU1Hr1G069990	chr1H:487882055–487,884,794	25		16	7
IIa	HvPUB4	HORVU2Hr1G068080	chr2H:478112149–478,116,380			2	
IIa	HvPUB5	HORVU2Hr1G084670	chr2H:612532269–612,544,059				
IIa	HvPUB6	HORVU2Hr1G107270	chr2H:711398163–711,399,326	2		8	3
IIa	HvPUB7	HORVU3Hr1G081300	chr3H:594390155–594,392,901	8			2
IIa	HvPUB9	HORVU3Hr1G113910	chr3H:689573848–689,580,280	8	1	2	11
IIa	HvPUB10	HORVU4Hr1G059610	chr4H:497914351–497,923,545	7	1	8	10
IIa	HvPUB11	HORVU5Hr1G021270	chr5H:102370756–102,375,777	20	2	22	12
IIa	HvPUB12	HORVU5Hr1G021280	chr5H:102438361–102,442,824	19	2	15	10
IIa	HvPUB13	HORVU5Hr1G059280	chr5H:463207931–463,210,666	69		2	4
IIa	HvPUB14	HORVU6Hr1G069010	chr6H:478128962–478,134,602	6		14	6
IIa	HvPUB15	HORVU6Hr1G072420	chr6H:503270970–503,276,564	9		6	
IIa	HvPUB16	HORVU6Hr1G073280	chr6H:507756329–507,760,902	18		13	9
IIa	HvPUB17	HORVU7Hr1G000780	chr7H:1309283–1,315,775	8	1	6	14
IIa	HvPUB18	HORVU7Hr1G047920	chr7H:162626044–162,632,315				
IIa	HvPUB19	HORVU7Hr1G061800	chr7H:290933777–290,936,462	10		3	
IIa	HvPUB20	HORVU7Hr1G121810	chr7H:654539257–654,541,940			3	2
IIa	HvPUB21	HORVU6Hr1G041430	chr6H:228200647–228,211,681	10		8	25
IIb	HvPUB22	HORVU2Hr1G067610	chr2H:473427629–473,435,416				
IIb	HvPUB24	HORVU3Hr1G083500	chr3H:603823686–603,825,481	2		2	4
IIb	HvPUB25	HORVU5Hr1G029950	chr5H:183287072–183,291,627	19	1	13	32
IIb	HvPUB26	HORVU5Hr1G059910	chr5H:467836849–467,839,703	2		2	
IIb	HvPUB28	HORVU7Hr1G039760	chr7H:105941903–105,943,737	5		2	
IIb	HvPUB29	HORVU7Hr1G040790	chr7H:111351005–111,354,169	3	1	3	2
IIb	HvPUB57	HORVU6Hr1G066870	chr6H:463530777–463,532,567				
IIb	HvPUB60	HORVU6Hr1G095130	chr6H:583093713–583,097,921	9			
III	HvPUB31	HORVU4Hr1G070330	chr4H:574972338–574,976,289				2
IV	HvPUB32	HORVU1Hr1G053270	chr1H:393963164–393,967,064	1		3	1
IV	HvPUB33	HORVU2Hr1G013130	chr2H:28662118–28,667,022	6			1
IV	HvPUB34	HORVU4Hr1G017550	chr4H:78074992–78,084,602	2	1	2	1
IV	HvPUB35	HORVU4Hr1G088650	chr4H:640685328–640,696,684		1	1	5
IV	HvPUB36	HORVU5Hr1G060580	chr5H:474627227–474,645,070	6			2
IV	HvPUB37	HORVU5Hr1G077700	chr5H:553639844–553,654,837				1
IV	HvPUB38	HORVU6Hr1G003590	chr6H:8039677–8,048,008			1	
IV	HvPUB39	HORVU6Hr1G039290	chr6H:203742583–203,750,519				
IV	HvPUB40	HORVU6Hr1G064130	chr6H:433376429–433,381,410		1		2
IV	HvPUB41	HORVU7Hr1G018750	chr7H:25113620–25,118,764	2		1	7
IV	HvPUB42	HORVU7Hr1G086580	chr7H:522444326–522,451,758	14		11	22
V	HvPUB8	HORVU3Hr1G089040	chr3H:627083327–627,148,340	17		7	14
V	HvPUB23	HORVU4Hr1G064070	chr4H:536981409–536,983,117	11	1		
V	HvPUB27	HORVU6Hr1G077120	chr6H:528582237–528,584,114		4	2	6
V	HvPUB30	HORVU7Hr1G046920	chr7H:155292351–155,294,342	6		2	6
V	HvPUB43	HORVU2Hr1G104640	chr2H:704314927–704,319,431	1		1	
V	HvPUB44	HORVU3Hr1G095960	chr3H:651507615–651,508,824				
V	HvPUB45	HORVU5Hr1G005830	chr5H:9430272–9,431,296				
V	HvPUB46	HORVU5Hr1G081160	chr5H:563433753–563,438,456				
V	HvPUB47	HORVU7Hr1G093780	chr7H:572254844–572,256,019				2
V	HvPUB48	HORVU0Hr1G003810	chrUn:17443362–17,445,805	13			8
V	HvPUB49	HORVU1Hr1G074180	chr1H:507187193–507,189,384	2			6
V	HvPUB50	HORVU2Hr1G074130	chr2H:535102978–535,105,576	10		10	12
V	HvPUB51	HORVU2Hr1G076470	chr2H:550697966–550,699,310				
V	HvPUB52	HORVU2Hr1G105720	chr2H:707375224–707,377,465	2			5
V	HvPUB53	HORVU2Hr1G123130	chr2H:754722899–754,723,432	1			
V	HvPUB54	HORVU4Hr1G066070	chr4H:550510764–550,513,664	2		21	13
V	HvPUB55	HORVU6Hr1G034250	chr6H:159856080–159,857,826			2	
V	HvPUB56	HORVU6Hr1G065480	chr6H:450926585–450,933,083	4		2	
V	HvPUB58	HORVU6Hr1G089560	chr6H:570052386–570,053,961	19			
V	HvPUB59	HORVU6Hr1G089590	chr6H:570107770–570,109,318	14			
V	HvPUB61	HORVU7Hr1G073100	chr7H:412252306–412,361,703	2			4
VI	HvPUB62	HORVU1Hr1G039050	chr1H:272999682–273,010,309	19		20	5
VI	HvPUB63	HORVU3Hr1G052520	chr3H:381485236–381,496,734	7		2	
VI	HvPUB64	HORVU4Hr1G083960	chr4H:627004063–627,010,259			10	1
VII	HvPUB65	HORVU1Hr1G002240	chr1H:4416074–4,418,966	4	1	1	10
VII	HvPUB66	HORVU7Hr1G076230	chr7H:445084899–445,090,758	7		11	3
X	HvPUB67	HORVU4Hr1G022990	chr4H:122902796–122,907,027	9		8	8

### Class I and class II U-box E3 ligases

**Fig. 2:**
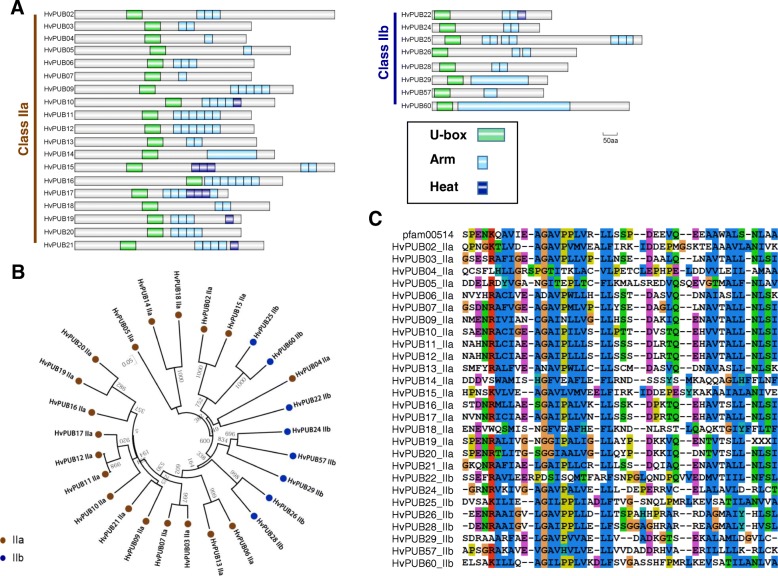
Domain structures and phylogenetic analysis of Class II genes in barley. **a**. Domain structures of 23 Class II *PUB* genes. Green box, U-box domain; sky blue box, ARM repeat domain; blue box, Heat domain. **b**. Phylogenetic analysis of 23 Class II *PUB* genes in barley. Brown dot, subclass a; blue dot, subclass b. **c**. Full-length amino-acid sequences of ARM repeat domain were aligned using the Clustal X2 software. The tree was constructed by neighbor-joining method after bootstrap analysis for 1000 replicates [11]

**Fig. 3:**
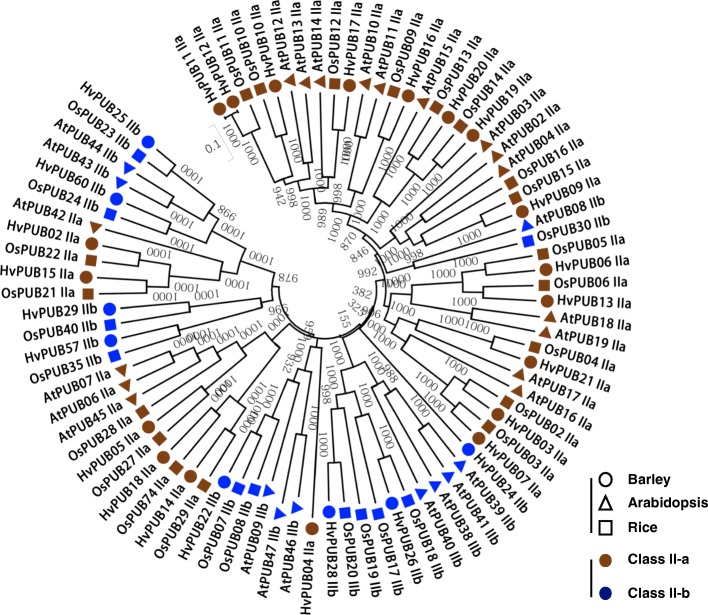
Phylogenetic tree of Class II genes in Arabidopsis, rice and barely. Brown color indicates Class II-a. Blue color indicates Class II-b. Triangle, Arabidopsis; Circle, barley; Square, rice

In Arabidopsis, rice and barley, the Class I E3 ligases are represented by a single protein member. The protein encoded by the barley gene *HvPUB1* contains a UFD2 domain and a U-box domain at the C-terminus like the *AtPUB1* and *OsPUB1* encoded proteins (Fig. 1b, c). For Class II, we found genes encoding 27 barley U-box proteins possessing repeated ARM/HEAT domains (Fig. 1c). The ARM domains of the 27 *Hv*PUB proteins were highly conserved with only minor variations in the consensus sequences with 11~48% identity and 32~68% similarity (Fig. 2c). When the full amino acid sequences and domain arrangement of the 27 proteins were compared, we recognized that the Class II proteins can be further assorted into two distinctive groups depending on the proximity of the U-box domain to the N-terminal: the one-fourth of the full length. (Fig. 2a). Class II-a contains 19 members with the U-box positioned near the center of each protein and a U-box N-terminal domain (UND) constituting the N-terminal part. All ARM repeats are positioned in the C-terminal half of the proteins. Class II-b contains 7 members with the U-box domain positioned close to the N-terminal and ARM repeats distributed over the remaining part of the protein sequence (Fig. 2a). Phylogenetic analysis of the barley proteins shows that all the Class II-b proteins share common ancestors with Class II-a genes in the same clades (Fig. 2b). Considering that all the proteins in Class II-b are absent in UND region, Class II-b proteins might have diverged from a Class II-a gene by merely losing the UND region. The opposite scenario is possible that a common ancestor of Class II protein might do not have UND region and Class II-a acquired the UND region later. However, the consensus sequences of the Class II-b ARM repeats are identical to those of Class II-a, implying that the II-a/II-b evolutionary branching has occurred after integration of the ARM repeats (Fig. 1c) and the branching happened in two different points (Fig. 2b). Therefore, it could be more rational to accept the first scenario to explain the branching of these subgroups. In our study, Arabidopsis has 27 *AtPUB* genes encoding Class II proteins possessed ARM/HEAT repeats. Based on the presence or absence of the UND domain, 17 belonged to Class II-a and 10 belonged to Class II-b (Additional file 1: Figure S1). In rice 30 of the *OsPUB* genes possessed ARM/HEAT repeats and were classified as Class II proteins and 19 belonged to Class II-a and 11 belonged to Class II-b (Additional file 1: Figure S2). The validity of Class II sub-grouping was confirmed by the phylogenetic analysis of all Class II PUB sequences from Arabidopsis, rice, and barley which revealed distinct Class II-a and Class II-b sub-groups (Fig. 3). In general, the Class II PUB sequences are highly conserved between all three species suggesting that all Class II-a and Class II-b sub-groups share common ancestors for all the tested species. This would imply that the Class II subgroups arose before the evolutionary specification of these species (Fig. 3).

### Class III, class IV, and class V U-box E3 ligases

**Fig. 4:**
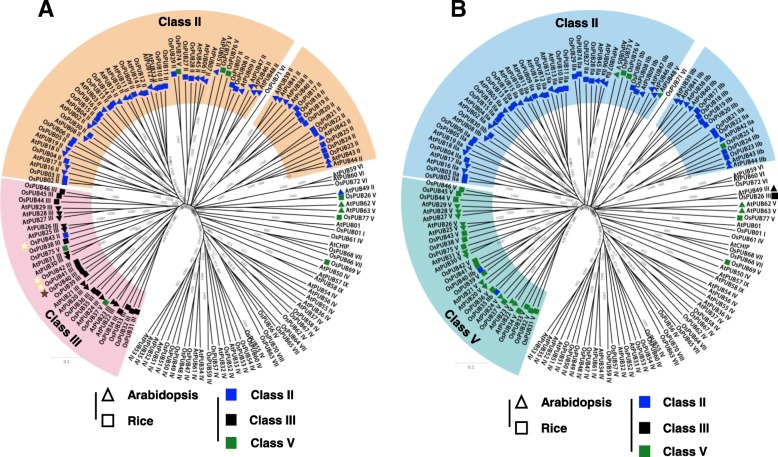
Phylogenetic analyses of *PUB* genes in Arabidopsis and rice. **a**. Phylogenetic analysis based on the classification category of previous studies [10]. In this phylogenic analysis, GKL-domain proteins were classified as Class III. Yellow asterisks indicated genes without ARM repeat domain in rice, *OsPUB41*, *OsPUB42*, *OsPUB43*. Red asterisk indicates *OsPUB40* with ARM repeat domain. **b**. Phylogenetic analysis based on the renaming category that was suggested in this study. Most of Class III genes in Arabidopsis and rice were renamed into Class V except *OsPUB35* and *OsPUB40* that were sorted into Class II

**Fig. 5:**
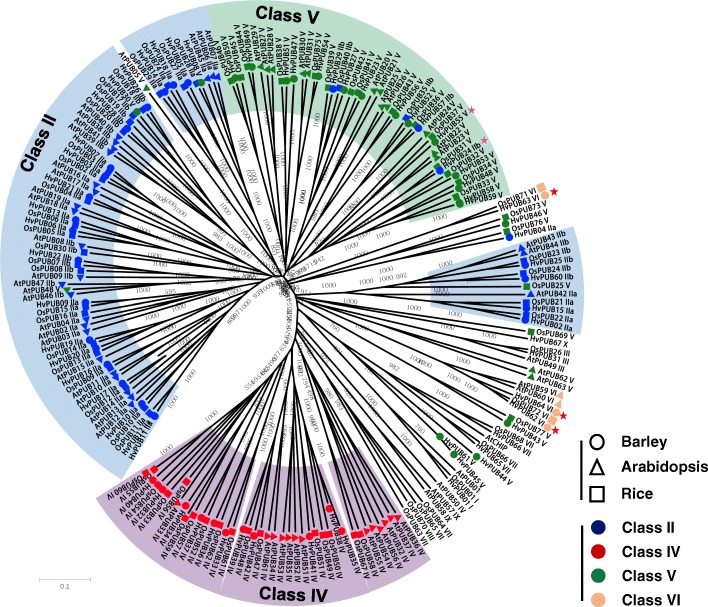
Phylogenetic analysis of *PUB* genes in Arabidopsis, rice, and barley. Class II genes were clustered and colored with blue. Class IV genes were clustered and colored with red. Class V genes were clustered and colored with green. Class VI genes were not clustered and indicated with incarnadine colored marks. Red asterisks indicate barley genes in Class VI. Pink asterisks show *HvPUB24* and *HvPUB55* gene pair

Previously, Zeng et al. suggested that Arabidopsis and rice Class III U-box proteins harbor a putative GKL-box domain in addition to the U-box domain [10] (Fig. 4a). However, we could not find any evidence that supports the functionality of the proposed GKL-box domain in eukaryotes. *Os*PUB40 has been reported as a protein harboring a GKL-box domain protein [10] but it contains a clear ARM repeat domain in our analysis. Considering that its clustered neighbours *Os*PUB41, *Os*PUB42, and *Os*PUB43 do not contain an ARM repeat domain, its presence in *Os*PUB40 was intriguing (Fig. 4a). This observation led us to search for the ARM repeat sequence of *Os*PUB40 in other Class III PUBs. Surprisingly, most of the rice Class III PUBs possess a domain which share high sequence homology to an ARM repeat domain albeit with slight aberrancies in the consensus sequences. Therefore, we redefined the GKL-domain as an ARM-like region. Likewise, most of the Class III PUB proteins in Arabidopsis also contained ARM-like regions (Additional file 1: Figure S3). However, ARM-like region cannot be considered as ARM repeats domain. Therefore, we suggest that Class III proteins in Arabidopsis and rice should be regrouped with the Class V proteins, which characterized by having no distinctive functional domains. In this context, 16 and 20 proteins of Arabidopsis and rice, respectively, were re-classified from Class III to Class V. Based on these criteria, 21 *Hv*PUBs genes were assigned to Class V (Fig. 1c, Fig. 4b). Our analyses of the PUB proteins in barley further revealed that the barley *HvPUB31* gene encoded a protein with a cyclophilin domain in addition to the U-box domain. This E3 ligase makes a distinct phylogenetic group joined by two orthologues proteins *At*PUB49 and *Os*PUB26 (Fig. 1b and c, Table 1, Fig. 5). Although distinctive, PUB proteins with a cyclophilin domain have not previously been considered as an independent class. Because the peptidyl prolyl isomerase activity of cyclophilin is well-defined in many proteins, we suggest that this type of PUB proteins are combined into a new Class III. Eleven *Hv*PUB proteins were found to contain a kinase domain in addition to the U-box. Class IV PUB proteins contain a PKc (Catalytic domain of the Serine/Threonine kinases) domain at the C-terminal (Additional file 1: Figure S4) and/or an STK_N (N-terminal domain of Eukaryotic Serine Threonine kinases) domain at the N-terminal. Following the previous classification in Arabidopsis and rice [10, 12], we assorted those genes into Class IV. Rice and Arabidopsis and hold 17 and 14 homologue Class IV *PUB* genes, respectively (Fig. 1b and c, Table 1, Fig. 5). We found that Class IV can be grouped into three subgroups; subgroup I has both kinase domains, while subgroup II has six genes with only PKc domain and subgroup III has five genes with only STK-n domain (Additional file 1: Figure S4). Phylogenic analysis, according to their whole sequence homology, not by the kinase domain compositions, showed that the subgroup III might be branched by simply losing the PKc domain, which only happened in Arabidopsis. Besides, subgroup II seemed to be randomly branched from subgroup I of rice and barley (Additional file 1: Figure S5).

### The other classes of U-box E3 ligases

Previously, *PUB* genes that encode proteins with a U-box at the N-terminal and a WD40 domain at the C-terminal were classified as Class VI [10]. We found that barley has three *HvPUB* genes encoding proteins belonging to Class VI (Fig. 1b and c, Table 1, Fig. 5). The domain organization in *Hv*PUB63 is different from *Hv*PUB62 and *Hv*PUB64 even though the three genes are assigned into Class VI (Additional file 1: Figure S6). We further identified two TRP domain containing *PUB* genes and assorted them as Class VII in barley (Fig. 1b and c, Table 1, Fig. 5). Arabidopsis and rice have a single and five members of Class VII. In Arabidopsis, two *At*PUB proteins have a MIF4G domain at the N-terminal region in addition to a U-box domain. These Arabidopsis specific genes were classified into Class IX (Fig. 1b and c, Table 1). Class VIII is specific for rice, containing one-member protein which contains a TRP domain and a kinase domain. In addition, our analysis revealed a barley specific U-box protein, *Hv*PUB67, which at the N-terminal region harbours a DJ-1 protein domain for thiamin biosynthesis. Because of the domain specificity, we assigned the DJ-1 containing PUB E3 ligase to a new class, Class X (Fig. 1b and c, Table 1, Fig. 5).

### Phylogenetic relationship of PUB proteins in Arabidopsis, rice, and barley

Our phylogenetic analysis of barley, rice and Arabidopsis showed that most PUB proteins have much closer phylogenetic relationships within their clustered orthologues from other species than their paralogues (Fig. 5). This distinctive grouping among the different classes of PUB proteins across species implied that PUB proteins have largely diversified from a common ancestor before the evolutionary branching of monocots and dicots. For instance, Class II and V proteins were highly clustered among Arabidopsis, rice and barley (Fig. 5) suggesting that Class II and Class V might share common ancestors which harbor an ARM repeat domain, then they diverged into two distinctive groups, resulting in one group maintaining an ARM repeat domain and the other losing the consensus sequences of the domain. Class IV proteins were also highly well clustered into three different clades (Fig. 5). Clade I consists of 6 Arabidopsis, 5 rice and 5 barley proteins, whereas Clade II has 1 Arabidopsis, 9 rice and 6 barley proteins. Clade III does not include barley proteins (Additional file 1: Figure S5). In the case of Class VI, two Arabidopsis proteins (*At*PUB59 and *At*PUB60), one rice protein (*Os*PUB72) and two barely proteins (*Hv*PUB62 and *Hv*PUB64) were well clustered in the clade (Fig. 5). *Hv*PUB63 is positioned in an independent clade together with *Os*PUB71, which is not related to Class VI. This result implied that the two different clades of Class VI also branched before the speciation of the three species. Furthermore, we found that several *Hv*PUB proteins belonging to Class V are more related to Class I and Class VII than to the largest clade of Class V. For instance, *Hv*PUB44, *Hv*PUB45, and *Hv*PUB61 fall into Class V based on the module content but form an independent sister taxon, in between Class I genes and Class VII genes. We speculate that the phylogenic distribution of these barley genes might be caused by losing a TRP or UFD encoding domain during their evolution. Similarly, several genes in Arabidopsis and rice may during evolution have lost the sequence encoding the ARM repeat domains. For instance, two Class V gene in Arabidopsis encoding *At*PUB5 and *At*PUB48 seemed to have branched from Class II genes. In rice, *OsPUB25* may have diverged from Class II genes. *HvPUB8* and *HvPUB30* genes also appear to have diverged from Class II genes (Fig. 5). In contrast, we observed that several rice and barley Class II genes may have been reverted from Class V. For example, *OsPUB35*, *OsPUB40*, *HvPUB24*, *HvPUB29*, and *HvPUB57* were clustered with Class V genes although these genes contain sequences encoding ARM repeats (Fig. 5). As shown here, the classification of PUB proteins in plants solely based on their defined domains imposes ambiguity in assigning the proteins into specific Classes and Clades. However, most of the PUB proteins in barley show similar phylogenetic distributions to those in Arabidopsis and rice, implying that *PUB* genes are evolutionarily well conserved across dicots and monocots.

### Expression profile of *HvPUB* genes in response to drought stress

**Fig. 6:**
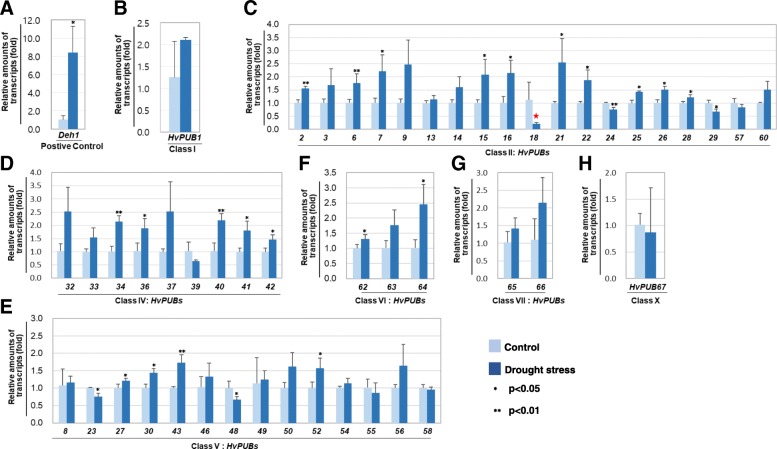
The expression profiles of *HvPUB* genes of class I (**a**), II (**c**), IV (**d**), V (**e**), VI (**f**), VII (**g**), X (**h**), and Dehydrin1 (**b**) in response to drought stress. Two-week-old seedlings were treated with drought stress for 24 h. *Dehydrin1* (*Deh1*) was used as a positive control. Three different biological samples were tested and four technical replicates used for the tests. Data were normalized to a reference gene, barley actin. Statistical significance was determined using Student’s t-test (*P < 0.05, **P < 0.01)

*PUB* genes are negatively or positively involved in plant responses to drought stress. The expression profiles of the majority of the identified barley *HvPUB* genes as response to drought stress were investigated by subjecting two-weeks-old barley seedlings to abrupt dehydration conditions. The onset of drought responses in the seedling plants was monitored as induced expression of the well-defined drought induced gene *Dehydrin1* (*Deh1*) (Fig. 6a). In the drought stress treated seedlings, *Deh1* expression increased 8-fold compared to the non-stressed control seedlings.

As shown in Fig. 6, the expression of the barley *HvPUB* genes were dynamically altered by the drought stress. A subset of the genes showed two-fold increase in expression when compared to the control plants. Significant changes in expression after drought stress were observed for *HvPUB* genes from Class II, Class VI and Class VI. Within Class II, expression of five genes, *HvPUB7, HvPUB9, HvPUB15, HvPUB16, HvPUB21* and *HvPUB22* were notably induced, whereas expression of *HvPUB18* was strongly reduced (Fig. 6c). Within Class IV, the expression of four genes, *HvPUB32, HvPUB34, HvPUB37,* and *HvPUB40* was induced by around two-fold (Fig. 6d). The expression of the *HvPUB64* gene from Class VI was increased over two-fold (Fig. 6f). In Class V and Class VII, none of the genes was significantly up-regulated by drought stress (Fig. 6g and e). The expression of 11 out of 67 barley *HvPUB* genes was at least two-fold induced in seedlings under the drought treatment and the expression of the gene *HvPUB18* was suppressed, supporting the strong involvement of the *PUB* gene family in plants drought stress responses (Fig. 6, Additional file 1: Figure S7).

### Expression profile of *HvPUB* genes in response to fungal infection

**Fig. 7:**
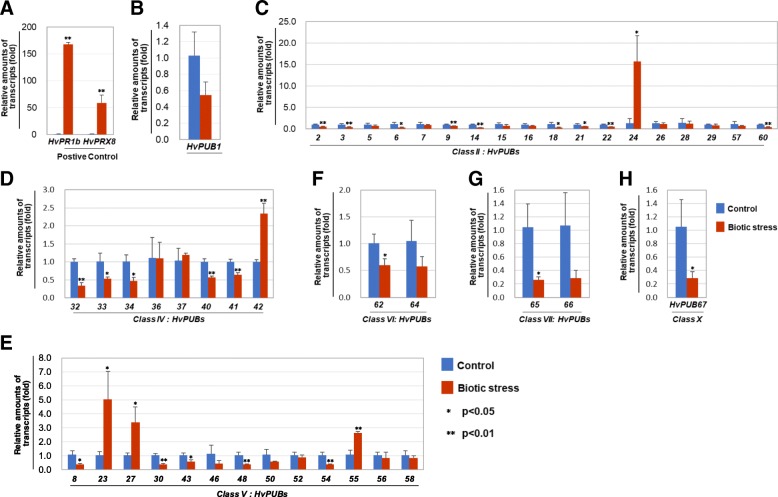
The expression profiles of *HvPUB* genes of class I (**b**), II (**c**), IV (**d**), V (**e**), VI (**f**), VII (**g**) and X (**h**), and positive control genes (A) in response to biotic stress. Two-week-old wild type barley seedlings were treated with powdery mildew for 16 h. *HvPR1b* and *HvPRX8* were used as positive controls. Three different biological samples were tested and four technical replicates used for the tests. Data were normalized to a reference gene, barley actin. Statistical significance was determined using Student’s t-test (*P < 0.05, **P < 0.01)

*PUB* genes are also known to play a regulatory role in plant responses to pathogen stress. Changes in the expression profile of *HvPUB* genes in response to fungal infection was investigated using barley powdery mildew as the experimental system. Two weeks-old barley seedlings were inoculated with *Blumeria graminis* f.sp. *hordei* (*Bgh*) spores and 18 h after inoculation, the expression of the two pathogen responsive marker genes *HvPR1b* and *HvPRX8* was monitored to ascertain the activation of pathogen stress responses. Expression of *HvPR1b* and *HvPRX8* was induced by around 170 and 60-fold, respectively, compared to uninfected control plants (Fig. 7a). Overall, the expression of the tested *HvPUB* genes was suppressed after fungal infection. Only expression of the Class II gene *HvPUB24*, the Class IV gene *HvPUB42* and the Class V genes *HvPUB23*, *27* and *55* showed a significant 2-fold or higher induction (Fig. 7c, d, and e). The strongest suppression of expression (more than 3-fold) was observed for the Class II genes *HvPUB6*, *14* and *18*, the Class IV gene *HvPUB32*, the Class V genes *HvPUB8*, *30*, *46*, *48* and *54*, the Class VII genes *HvPUB65* and *66* and the Class X gene *HvPUB67* (Fig. 7, Additional file 1: Figure S8). In general, the observed changes in expression were not only larger after pathogen stress than the changes observed following drought stress, but they also involved different *HvPUB* genes. Only *HvPUB18* was regulated similarly, i.e. suppressed expression in response to both stress conditions, implying that it might be a negative regulator for both types of stress.

## Discussions

In Arabidopsis and rice, *PUB* genes are known to play important roles in the plant responses against abiotic and biotic stresses. Functional analysis has revealed detailed molecular mechanisms involving PUB proteins in relation to abiotic stresses and biotic stresses [6]. However, the *PUB* gene family in barley has so far not been investigated or implicated in barley stress responses. Our analysis identified 67 *PUB* genes in the barley genome. Based on domain structures and evolutionary relationship, the Arabidopsis, rice and barley *PUB* genes were categorized into ten different classes. This classification represents a refinement of the previously reported classifications of *PUB* genes in Arabidopsis and rice [10, 12]. Arabidopsis ARM repeat containing PUB proteins have previously been divided into two sub-groups based on the presence or absence of a UND (U-box N-terminal domain) region [7]. We adapted this in our classification as Class II-a with UND and Class II-b without the UND region. The UND region does not affect the E3 ligase activity, but was shown to determine the ubiquitination specificities of *At*PUB18 [37]. No function of the UND region has yet been reported for rice or barley. Based on the phylogenetic analysis it seems that Class II-b *PUB* genes from the analyzed species have evolved from common ancestral genes rather than from Class II-a *PUB* genes. Previous studies suggested that 28 *PUB* genes in rice and 29 *PUB* genes in Arabidopsis belong to Class II [10]. However, bioinformatic analysis using the most recent sequence data, we redistributed several of the previously assigned Class II genes of Arabidopsis and rice into other classes. For instance, we could not confirm the presence of sequences encoding ARM/HEAT repeats in two Arabidopsis (*AtPUB05* and *AtPUB48*) and three rice genes (*OsPUB25*, *OsPUB73*, and *OsPUB76*). Accordingly, these genes were therefore transferred into Class V among the other PUB proteins without extra subdomains (Fig. 4, Additional file 1: Figure S1 and S2).

Zeng et al. [10] identified a motif with highly conserved glycine, lysine/arginine and leucine rich residues in the C-terminal part of several Arabidopsis and rice PUB proteins and named this region as a GKL-domain. Based on the presence of this putative domain, these PUB proteins were placed in Class III, independent on the presence of other domains. However, our analyses revealed that the GLK domain that formed the basis for the grouping of certain PUB proteins in Class III exhibited a high degree of sequence similarity to the ARM repeats. Re-analysis of the Arabidopsis and rice PUB proteins assigned to Group III by Zeng et al. demonstrated that *At*PUB21, *At*PUB30, *Os*PUB39, and *Os*PUB40 contain obvious ARM repeat domains. In the other PUB proteins classified to Group III, a few of the conserved residues of the ARM domain were missing. We therefore decided not to classify these PUBs with ARM-like region to Class II but instead assigned these sequences to Class V for which the common denominator is the absence of other domains than the U-box (Additional file 1: Figure S3). In the study of Zeng et al., *At*PUB49 and *Os*PUB26 were classified as Class II and Class V proteins, respectively. We found that both proteins contain a cyclophilin domain in addition to the U-box domain and discovered that the barley protein (*Hv*PUB31) also harbored such a domain. We argue that the presence of a cyclophilin domain with peptidyl prolyl isomerase activity is so significant that it justifies the assignment of these proteins to a re-defined Class III. Our analysis also showed that five Arabidopsis genes (*AtPUB36*, *AtPUB37*, *AtPUB54*, *AtPUB55*, and *AtPUB56*) previously classified to Class V possess a clear serine/threonine kinase domain [24, 26]. Therefore, we re-classified these genes into Class IV (Fig. 5, Additional file 1: Figure S4). Compared to the classification of PUB proteins in Arabidopsis and rice, two classes (Class VIII and Class IX) are absent in barley. Rice *Os*PUB70 harbors a TRP as well as a kinase domain and is classified as Class VIII, while two Arabidopsis PUB proteins, *At*PUB57 and *At*PUB58, with MIF4G domains are classified as members of Class IX. Instead, barley has a unique U-box E3 ligase, *Hv*PUB67 which contains a DJ-1 domain with a U-box domain and is the only member of Class X. Interestingly, searching other plant genomes we could not find a ortholog of this protein. This suggests that *Hv*PUB67 is a unique barley protein.

Our investigation showed that most barley *HvPUB* genes were regulated in response to fungal attack by *Bgh* or in response to drought (Figs. 6 and 7). Five *HvPUB* genes were up regulated in response to *Bgh* attack, whereas, expression of the majority of the remaining *HvPUB* genes were down regulated in response to *Bgh*. In response to the imposed drought treatment, 11 *HvPUB* genes were activated and only one was down regulated. Except for the down regulation of *HvPUB18* under both biotic and drought stress, we did not observe any overlap in or contrasting regulation of the *HvPUB* genes, although a dual role of both abiotic and biotic stresses has been reported for many of the functionally characterized Arabidopsis and rice *PUB* genes [18, 26]. Arabidopsis *AtPUB20* (alternatively known as *AtCMPG1*) is immediately induced by an elicitor, indicating a role in the early steps of pathogen-host interactions in *Arabidopsis* [38]. In tobacco and tomato, *CMPG1* genes orthologous to *AtPUB20* are induced by elicitor and wounding treatments in a typical *ACRE* gene expression pattern. In tobacco, a deficiency in *NtPUB20* gene expression compromises the immunity of the plants [39]. *At*PUB20 is able to directly interact with the G-protein b-subunit, AGB1, but it is unclear whether this protein-protein interaction is relevant to plant defense [40]. Functional characterization of the orthologue *CMPG-V* from *Haynaldia villosa* L., a diploid wheat relative, showed that this *PUB* gene was induced in leaves and stem of *H. villosa* upon inoculation with the wheat powdery mildew fungus *Blumeria graminis* f. sp. *Tritici.* Over-expression of *CMPG1-V* in susceptible wheat lead to improved broad-spectrum powdery mildew resistance [35]. Our analysis showed that *HvPUB23* and *HvPUB27* are closely related to *AtPUB20* and *CMPG1-V*, with *Hv*PUB27 sharing more than 90% homology to CMPG1-V at the amino acid sequence level. In agreement with this observation, our fungal infection studies demonstrate that *HvPUB23* and *HvPUB27* were identified among the five genes showing induced expression after *Bgh* inoculation (Additional file 1: Figure S8), strongly implying a role of these *HvPUB* genes in barley powdery mildew resistance. Arabidopsis *AtPUB22*, *AtPUB23*, and *AtPUB24* function as negative regulators of pathogen-associated molecular patterns (PAMPs)-triggered responses. Upon encountering pathogens, plants recognize PAMPs by pattern recognition receptors [26]. Ubiquitination and vesicle trafficking have been linked to the regulation of immune signaling. In the signaling, Exo70B2, a subunit of the exocyst complex is required for both immediate and later responses [41]. AtPUB22 mediates the ubiquitination of Exo70B2 and its subsequent degradation by the 26S proteasome to attenuate PAMP-induced signaling [41]. Two close relatives in barley, *HvPUB24* and *HvPUB55* were up-regulated by fungal infection (Fig. 7c and e). Those genes might share a similar function as regulators of PAMP-triggered responses, as both were found to be induced following *Bgh* inoculation. Furthermore, a recent study suggested that *HvPUB15*/*ARM1* gene pair is a case of gene neo-functionalization after a non-tandem, partial gene-duplication event that gained a role in quantitative resistance against *Bgh* and maybe other pathogenic fungi [42]. Although *HvPUB15* was not significantly up-regulated by *Bgh* infection in our study, the non-tandem and partial gene-duplication of ARM domain in *HvPUB24*/*HvPUB55* gene pair could be another case of neo-functionalization for boosting pathogen tolerance (Fig. 5).

*At*PUB22 and *At*PUB23 play an essential role in drought responses. Mutations in these two genes have led to increased tolerance against drought stress relative to wild-type plants [18]. *At*PUB22 and *At*PUB23 conjugate ubiquitins to the 19S proteasome regulatory particle (RP) subunit RPN6, resulting in its degradation and altered activity of the 26S proteasome in response to drought stress [43]. In our study, the expression of barley *HvPUB24* was rather down-regulated and *HvPUB55* was not notably affected by drought conditions (Fig. 6). *AtPUB17* has been implicated as a positive regulator of plant defense and stress signaling responses [32], whereas the closely related rice orthologues *OsPUB2/3* were shown to play a role as positive regulators of temperature stress [21]. Barley has four orthologue genes, *HvPUB3, HvPUB7*, and *HvPUB21*, with *HvPUB3/7* sharing more than 80% homology at the amino acid level to *OsPUB2/3*. Expression of the *HvPUB3*, *HvPUB7* and *HvPUB21* genes were induced in response to our drought treatment, again suggesting a potential role like *Os*PUB2/3 in abiotic stress responses. Wheat *TaPUB1* is up-regulated in response to drought and constitutive over-expression of *TaPUB1* in tobacco enhanced drought tolerance in the transgenic plants, most likely caused by increased antioxidant capacity mediated by *Ta*PUB1 [34]. Wheat *Ta*PUB1 contains a WD40 domain and shares high homology with Arabidopsis, rice and barley Class VI PUB proteins. The Arabidopsis orthologues are *At*PUB59/60, also known as *MAC3A* and *MAC3B*, which in Arabidopsis are members of the MOS4-Associated Complex (MAC) that function redundantly in the regulation of plant innate immunity [44], but no role for *At*PUB59/60 in drought or other abiotic stresses has been reported. However, *Hv*PUB64 shares 96% homology to *Ta*PUB1 and is among most highly induced barley *HvPUB* genes in response to drought, implying a similar role of *HvPUB65* in mediating drought tolerance in barley as *TaPUB1* in wheat.

## Conclusions

From the current knowledge of the molecular and biological functions of *PUB* genes it seems that they possess a high degree of conserved functions in plant responses to biotic and abiotic stresses across species. Our data show that many barley *PUB* genes are also involved in responses against pathogen and drought stresses as negative or positive regulators. Although we showed that many barley *PUB* genes are transcriptionally regulated by the stresses, further investigations are needed to understand the molecular functions of barley *PUB* genes and their target proteins to shed further light over stress-response mechanisms in barley.

## Methods

### Plant materials and stress treatments

Barley (*Hordeum vulgare* L) cv. Golden Promise was from University of Copenhagen, Department of Plant Environmental Sciences, Denmark. It used for drought and pathogen treatments. Plants were grown in a growth chamber at 20 °C with a photoperiod of 16 h (200 μmol m^− 2^ s^− 1^).

### Drought treatment

Plants were grown in vermiculite for 2 weeks. The drought treatment was initiated by careful removal of the vermiculite attached to the roots and wrapping the roots in dry soft tissue paper to avoid any influence of light. Sampling took place when the leaves were observed to lose turgor pressure, approximately 24 h after imposure to the drought stress. Control plants were kept in watered in vermiculite.

### Powdery mildew infection

Plants were grown in a mixture of peat with granulated clay (Pindstrup substrate no. 6; Pindstrup Mosebrug A/S). Spores from *Blumeria graminis* f. sp. *hordei* (*Bgh*) Race A6 were inoculated onto the adaxial side of the second leaf with a density of 80 conidia mm^− 2^. Samples were taken 18 h after inoculation (hai).

### Sequence analysis

Barley *HvPUB* genes were predicted from the barley genome sequence [45], using the available Pfam and InterPro domain predictions [46, 47], blast searches with known *At*PUB and *Os*PUB genes and by using a Hidden Markov Model (HMM) built from an alignment of the *At*PUB and *Os*PUB U-box domains predicted by SMART program (http://smart.embl-heidelberg.de/). Conserved domains of the potential *Hv*PUB proteins were analyzed and confirmed using NCBI CDD (Conserved Domain Database) and InterPro protein domain predictions. Full length cDNA and EST sequences matching the predicted *Hv*PUB genes were identified by BLAST searches and their expression patterns assessed using the Barley Gene Expression Database (http://barleyflc.dna.affrc.go.jp/bexdb/) [48] and NCBI. Analysis of alignment and phylogenetic tree of full length or U-box domain sequences was performed by MEGA7 [49] or CLC workbench software.

### Phylogenetic analysis

The multiple sequence alignments of barley u-box E3 ligase proteins were performed to construct a phylogenetic trees by Clustal X version 2.1 [11]. All phylogenetic trees were constructed according to the using default settings and 1000 bootstrap replications were used to estimate the accuracy of trees.

### Real-time qRT-PCR analyses

Total RNA was isolated from drought-treated and powdery mildew inoculated two-week-old barley seedlings using Spectrum™ Plant Total RNA Kit (Sigma). First-strand cDNA was synthesized from 2 μg total RNA using the iScript™ cDNA Synthesis Kit (Bio-Rad). Real-Time qRT-PCR was performed using the CFX384 Touch™ Real-Time PCR Detection System (Bio-Rad) with DyNAmo Flash SYBR Green qPCR Kit (Thermo). Each qRT-PCR reaction was performed in technical triplicate using the barley u-box primers in this analysis (Additional file 2: Table S1.) All qRT-PCR data was analysed by using CFX Manager Version 3.1 software (Bio-Rad). Relative gene expression fold was calculated by delta-delta Ct method and the error bars means the ± standard deviation over technical triplicates of Cq values.

## Additional files

Additional file 1: **Figure S1.** Domain structures and phylogenetic analysis of Class II genes in Arabidopsis. A. Phylogenetic analysis of 27 Class II *PUB* genes in barley. Brown dot, subclass a; blue dot, subclass b. B. Full-length amino-acid sequences of ARM repeat domain were aligned using the Clustal X2 software. The tree was constructed by neighbor-joining method after bootstrap analysis for 1000 replicates [1]. C. Domain structures of 27 Class II *PUB* genes. Green box, U-box domain; skyblue box, ARM repeat domain; blue box, Heat domain. **Figure S2.** Domain structures and phylogenetic analysis of Class II genes in rice. A. Phylogenetic analysis of 25 Class II *PUB* genes in barley. Brown dot, subclass a; blue dot, subclass b. B. Full-length amino-acid sequences of ARM repeat domain were aligned using the Clustal X2 software. The tree was constructed by neighbor-joining method after bootstrap analysis for 1000 replicates [1]. C. Domain structures of 25 Class II *PUB* genes. Green box, U-box domain; skyblue box, ARM repeat domain; blue box, Heat domain. **Figure S3.** Class III genes in Arabidopsis and rice, those were converted to new classes, Class II and Class V. **Figure S4.** Schematic domain structures of Class IV genes in Arabidopsis, rice and barley. **Figure S5.** Phylogenetic analysis of Class IV *PUB* genes in Arabidopsis, rice and barely. **Figure S6.** Domain structures of ClassVI *PUB* genes in Barley. **Figure S7.** The expression profiles of HvPUB genes in response to drought stress. A. *HvPUB* genes are up-regulated by drought stress. B. *HvPUB* genes are down-regulated by drought stress. **Figure S8.** The expression profiles of HvPUB genes in response to biotic stress. A. *HvPUB* genes are up-regulated by biotic stress. B. *HvPUB* genes are down-regulated by biotic stress. (DOCX 3262 kb)

Additional file 2: **Table S1.** List of primers and sequence information. (DOCX 17 kb)

## Abbreviations

ABA: abscisic acid
ARM: Armadillo
CHIP: Carboxyl terminus of HSC70-interacting protein
*Hv*: *Hordeum vulgare*
PAMPs: pathogen-associated molecular patterns
PUB: PLANT U-box
UND: U-box N-terminal domain
UPS: ubiquitin proteasome system
