# Supplementary material for: Classification of barley U-box E3 ligases and their expression patterns in response to drought and pathogen stresses
Source: BMC Genomics. 2019 Apr 29;20:326. doi: 10.1186/s12864-019-5696-z (PMC6489225; doi:10.1186/s12864-019-5696-z)
Supplement: Supplementary file 1 — Figure S1. Domain structures and phylogenetic analysis of Class II genes in Arabidopsis. A. Phylogenetic analysis of 27 Class II PUB genes in barley. Brown dot, subclass a; blue dot, subclass b. B. Full-length amino-acid sequences of ARM repeat domain were aligned using the Clustal X2 software. The tree was constructed by neighbor-joining method after bootstrap analysis for 1000 replicates [1]. C. Domain structures of 27 Class II PUB genes. Green box, U-box domain; skyblue box, ARM repeat domain; blue box, Heat domain. Figure S2. Domain structures and phylogenetic analysis of Class II genes in rice. A. Phylogenetic analysis of 25 Class II PUB genes in barley. Brown dot, subclass a; blue dot, subclass b. B. Full-length amino-acid sequences of ARM repeat domain were aligned using the Clustal X2 software. The tree was constructed by neighbor-joining method after bootstrap analysis for 1000 replicates [1]. C. Domain structures of 25 Class II PUB genes. Green box, U-box domain; skyblue box, ARM repeat domain; blue box, Heat domain. Figure S3. Class III genes in Arabidopsis and rice, those were converted to new classes, Class II and Class V. Figure S4. Schematic domain structures of Class IV genes in Arabidopsis, rice and barley. Figure S5. Phylogenetic analysis of Class IV PUB genes in Arabidopsis, rice and barely. Figure S6. Domain structures of ClassVI PUB genes in Barley. Figure S7. The expression profiles of HvPUB genes in response to drought stress. A. HvPUB genes are up-regulated by drought stress. B. HvPUB genes are down-regulated by drought stress. Figure S8. The expression profiles of HvPUB genes in response to biotic stress. A. HvPUB genes are up-regulated by biotic stress. B. HvPUB genes are down-regulated by biotic stress. (DOCX 3262 kb) [file 12864_2019_5696_MOESM1_ESM.docx]

**Additional file 1**

Figure S1

Figure S2

Figure S3

Figure S4

Figure S5

Figure S6

Figure S7


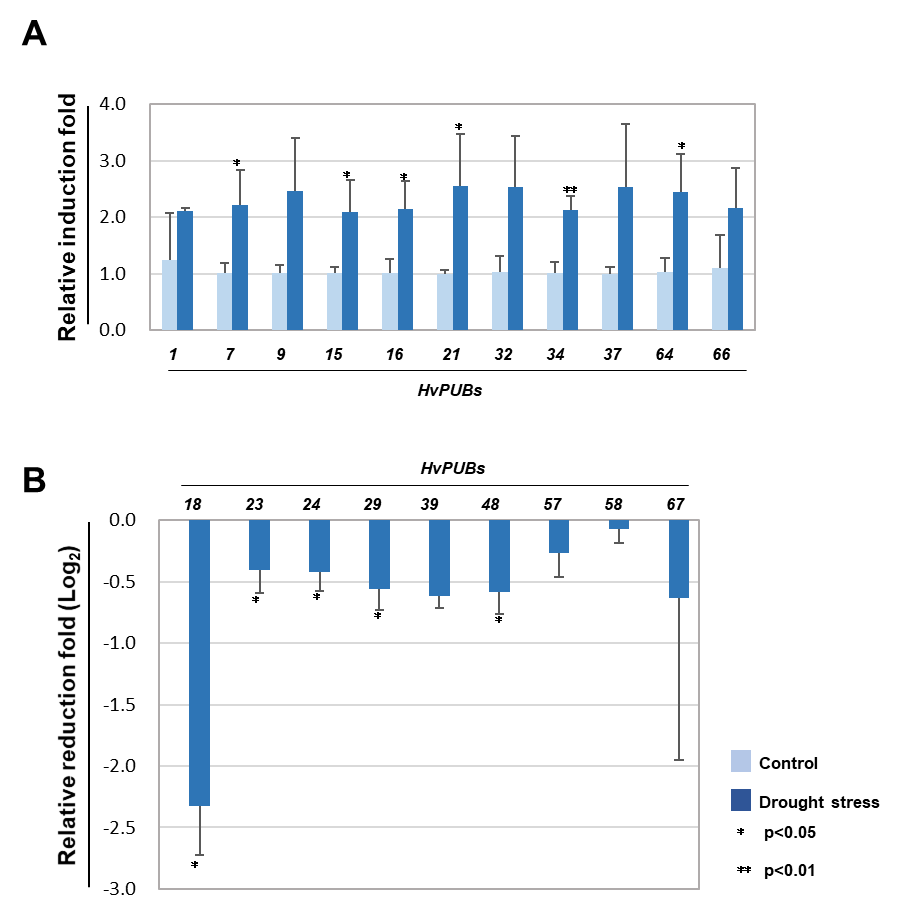


Figure S8


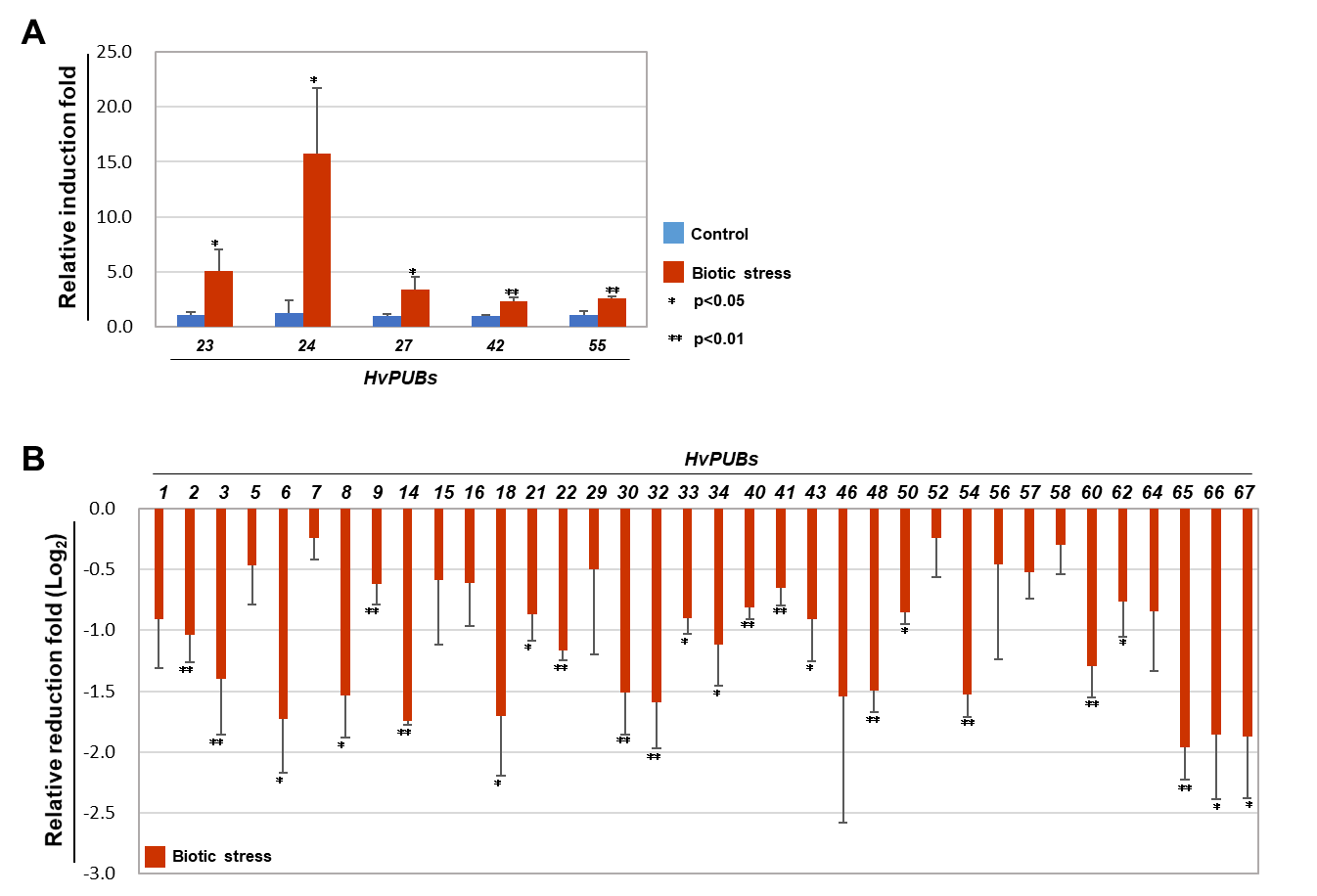


Figure S1. Domain structures and phylogenetic analysis of Class II genes in Arabidopsis. A. Phylogenetic analysis of 27 Class II *PUB* genes in barley. Brown dot, subclass a; blue dot, subclass b. B. Full-length amino-acid sequences of ARM repeat domain were aligned using the Clustal X2 software. The tree was constructed by neighbor-joining method after bootstrap analysis for 1,000 replicates [1]. C. Domain structures of 27 Class II *PUB* genes. Green box, U-box domain; skyblue box, ARM repeat domain; blue box, Heat domain.

Figure S2. Domain structures and phylogenetic analysis of Class II genes in rice. A. Phylogenetic analysis of 25 Class II *PUB* genes in barley. Brown dot, subclass a; blue dot, subclass b. B. Full-length amino-acid sequences of ARM repeat domain were aligned using the Clustal X2 software. The tree was constructed by neighbor-joining method after bootstrap analysis for 1,000 replicates [1]. C. Domain structures of 25 Class II *PUB* genes. Green box, U-box domain; skyblue box, ARM repeat domain; blue box, Heat domain.

Figure S3. Class III genes in Arabidopsis and rice, those were converted to new classes, Class II and Class V.

Figure S4. Schematic domain structures of Class IV genes in Arabidopsis, rice and barley.

Figure S5. Phylogenetic analysis of Class IV *PUB* genes in Arabidopsis, rice and barely.

Figure S6. Domain structures of ClassVI *PUB* genes in Barley.

Figure S7. The expression profiles of HvPUB genes in response to drought stress. A. *HvPUB* genes are up-regulated by drought stress. B. *HvPUB* genes are down-regulated by drought stress.

Figure S8. The expression profiles of HvPUB genes in response to biotic stress. A. *HvPUB* genes are up-regulated by biotic stress. B. *HvPUB* genes are down-regulated by biotic stress.

**Supplementary references**

1. Larkin MA, Blackshields G, Brown NP, Chenna R, McGettigan PA, McWilliam H, Valentin F, Wallace IM, Wilm A, Lopez R *et al*: **Clustal W and Clustal X version 2.0**. *Bioinformatics (Oxford, England)* 2007, **23**(21):2947-2948.

2. Zeng LR, Park CH, Venu RC, Gough J, Wang GL: **Classification, expression pattern, and E3 ligase activity assay of rice U-box-containing proteins**. *Molecular plant* 2008, **1**(5):800-815.
