# Supplementary material for: Classification of barley U-box E3 ligases and their expression patterns in response to drought and pathogen stresses
Source: BMC Genomics. 2019 Apr 29;20:326. doi: 10.1186/s12864-019-5696-z (PMC6489225; doi:10.1186/s12864-019-5696-z)
Supplement: Supplementary file 2 — Table S1. List of primers and sequence information. (DOCX 17 kb) [file 12864_2019_5696_MOESM2_ESM.docx]

Additional file 2: Table S1. **List of primers and sequence information**

| **HvPUB No.** | **Forward primer (5'→3')** | **Reverse primer (5'→3')** |
| --- | --- | --- |
| HvPUB01 | ACACGTCGTCCAGCTGATTT | TGCCAGATCTTCCTGCGTTT |
| HvPUB02 | CAACGTGATGCACATAGCCG | CACCTGTTGCTCCCAAATGC |
| HvPUB03 | TGCCCCATCTCTCTCGATCT | GTTCTTGAGCGCCTTGTTGG |
| HvPUB04 | AGCAGTGCTCATTCCTGCAT | CTCCTTGTTGGGGCTGTAGG |
| HvPUB05 | CTTGAAAGAGCCCAGGCAGA | ACGAAGGACTTGAGCACTGG |
| HvPUB06 | GTCCATCAAGCGGTGGATCA | CTTGCTGCTTTGCTGTTCGT |
| HvPUB07 | ATATCATGAGCTCCGGCACG | CGCACCAGGTGGACTAACTT |
| HvPUB08 | AGCGAATCAGGCACATTGGA | CCTTGCTTCCTCGTCGTCTT |
| HvPUB09 | CTGGAGAAGTTGCTGACGGT | AAATTGCCCTCTCATGCGGA |
| HvPUB13 | GAGTACGCCGAGGAGATTGG | AGACCGTAGAGGCTGACCAT |
| HvPUB14 | AGACACTGGTGAGCGGAATG | GGCTTGTGGCACTTTTCAGG |
| HvPUB15 | TTGTGTGCATGCTCAAGTGC | TGCTAAGTGCCTCCATTGGG |
| HvPUB16 | CATAGCCAAGGCTCACACCA | GGTATTTGAGCACCCAGCCT |
| HvPUB18 | AGAGCATTGCATCGGTTCCA | CAAGTGCAGGGATGACACCT |
| HvPUB19 | GAAGGTCCAGGAGAACACGG | GCATGGACAGGCTGAAGAGT |
| HvPUB20 | ATCCAGGACAACACGGTCAC | CACTGCCGTTCTTGAGGACT |
| HvPUB21 | ACCTCTCAACGCATGCAGAA | TCATCAGCAGAGCCAAAGCA |
| HvPUB22 | TGCTCCAAGCAATGAGGGAG | CAAGAGAAGCCAGTGAGCCA |
| HvPUB23 | CAGGATCTTGGGATCGCCTC | GCAGATGGGAGCCTTGATGA |
| HvPUB24 | CAAGAAGGTACTGCGGGTGT | CGCCTTCTCCTTGGTCTTGA |
| HvPUB25 | CCAACGCGGGATTGTGTTTT | ACTTCTCCACCACCTTCCCT |
| HvPUB26 | GTCGTCCTCGGAGATCACAC | CACCTCTCCATCTTCGTCCG |
| HvPUB27 | GTCGCTAGAATCCGTCGTGT | ATATGGCGGTGGTCTTGGAC |
| HvPUB28 | TCCGGACAAGAAACCGCTAC | TCCGCATCTCTTTGCTCTCC |
| HvPUB29 | CCTCGTCACGGCATACTACC | CTTCTCGCTCGTCCCCTTG |
| HvPUB30 | GCCTCCACTTCTGTCACTCC | ATGGAGAGCAGGTTCTTGGC |
| HvPUB32 | GGAAGCAAGTTCCACGCAAG | CGAGCTCCATGATCCCCTTC |
| HvPUB33 | ACTGACTGGTAAACGGCCTG | CTCAAGCCCAAACTCCGCTA |
| HvPUB34 | CGTTGTTGAGTCCCTCGTCA | TGGCTTCCGCTTCATAGGTG |
| HvPUB35 | TGAAAAAGGGCACACCACCT | TGAGGAGGACGTTTTCAGGC |
| HvPUB36 | GAGGCGTACGAAGAGACTCG | CCTCGTCAGACTCTCCTCCA |
| HvPUB37 | TTCTGATGCGGTTGCTGACT | CGCTGCTTTCGTTCATACCG |
| HvPUB39 | TGCAGGACATCCTCAAGCAG | AGAGCGTCTCAGCCTCTGTA |
| HvPUB40 | AGCGCATAACCGAGATGGAG | TGGTTATCGCGGCATCTCTC |
| HvPUB41 | ATGGGAGCATATCCAAGCGG | GCTCCTGCTGTAACTGCTGA |
| HvPUB42 | CTGAGGAGCCAGTTGCAAGA | AGCAGCACTTGCAGAAAAGC |
| HvPUB43 | TCGTTGCTGTTCTGCTGCTA | TCTCAGAGACTCCGTGAGGG |
| HvPUB46 | GACAAGTGGCCAGAGCTGAT | TGTTGGGCCTGTGTATGGAC |
| HvPUB48 | TGTTCGGCGCTGTAGTTGAT | CGCTTTGAGAAGCTGGATGC |
| HvPUB49 | AAGAAAGCCGTCATCACCGT | GGATCGACTCGAGAACCACG |
| HvPUB50 | ACAAGTACAAGGACCGCAGG | GTGGTCTTGGAGTCGTAGCC |
| HvPUB52 | CTGTTCCTCTGCCCGATCTC | TTGAAGAACCACCGCTCGAT |
| HvPUB54 | GCCAAGCTTCTCCTGGTGAT | TTGGTGAGCTTGCATTTCGC |
| HvPUB55 | TGATGTCCCCGACTTCTTGC | TCTTGCCGCACGACGATAAT |
| HvPUB56 | CCAATCTCGCTGGAGCTCAT | ACCATTCTTGGATGAGGCGG |
| HvPUB57 | GTGCTCGAGGATATGGTGCT | ATCCTTGAGATCGGTGGGGA |
| HvPUB58 | TGCTGCTGGTTCTACAGGTG | GCACTTGTAAGCATGGCGAG |
| HvPUB60 | CCGTGCGCACCATCATAAAG | GACCAGCAGGAGTATCGCTC |
| HvPUB62 | TACCGCTGAGGTTGAAGCTG | CTGTGAGAGGCAAGACCCAG |
| HvPUB63 | CATGATGCACACGCTTCTCG | TATCTTCCTCGGCTCCACCA |
| HvPUB64 | TCGCTGTTGCTGGTTCTGAT | TCTCCAGGGAGCCCGAATAA |
| HvPUB65 | GCACTCTACCCAGCGAGTTT | CCTGGCGTATCGGCAAGTAT |
| HvPUB66 | CAGGAAAGCTGCAAAGGCTG | TTGGTGTGGTTCCAGTGCTT |
| HvPUB67 | AGAGAAGTTTGCCGCACGTA | GCCACACAGTGGACAGGTAA |
| ACTIN | TCGAGCACGGTATCGTAAGC | ATAGCGACGTACATGGCAGG |
| HvPrx8 | GGCATGGAACAAAACGCTAT | GAGTATGTCGGCACAGGAG |
| HvPR1b | CCGCAGGACTACGTATCACC | TTGCAGTCGTTGATCCTCTG |
| HvDhn1 | TCGCAACAGATCAGCACACT | TACTCCTCCACCTTGTCGGT |
